# Using Sport to Build Inclusion Between Mainstream and Special Schools for Students with Intellectual Disabilities in Eastern Europe

**DOI:** 10.3390/ijerph23020249

**Published:** 2026-02-17

**Authors:** Roy McConkey, Sabine Menke, Eva Gazova, Emilia Ispas, Joanna Styczeń-Lasocka

**Affiliations:** 1 Institute of Nursing and Health Research, Ulster University, Belfast BT1 6DN, UK; 2 Special Olympics Europe Eurasia, Dublin 2, D02 X3P2 Dublin, Ireland; smenke@specialolympics.org; 3 Special Olympics Slovakia, Trnavská cesta 37, 831 04 Bratislava, Slovakia; gazova@specialolympics.sk; 4 Special Olympics Romania, Soseaua Alexandriei Nr. 96, 077025 Bragadiru, Romania; e.ispas@specialolympics.ro; 5 Special Olympics Poland, Ul. Leszno 21, 01-199 Warszawa, Poland; j.styczen@olimpiadyspecjalne.pl

**Keywords:** sports, intellectual disabilities, school, mainstream, special schools, Special Olympics, public health

## Abstract

**Highlights:**

**Public health relevance—How does this work relate to a public health issue?**

- Children with intellectual disabilities experience poorer health and emotional wellbeing which is compounded by their social exclusion.
- Segregated schooling limits their opportunities for inclusion with their peers in formal and informal health promoting activities, such as sports.

**Public health significance—Why is this work of significance to public health?**

- A model for bridging specialist and mainstream provision has been developed that could have wider implications beyond education.
- The focus is on building partnerships at a local level through committed personal relationships.

**Public health implications—What are the key implications or messages for practitioners, policy makers and/or researchers in public health?**

- Invest in local communities and trust them to build initiatives tailored to their needs and culture.
- Public health initiatives should assess and address the social exclusion of marginalized groups.

**Abstract:**

Children with intellectual disabilities experience social exclusion in all countries of the world. This is compounded too by their attendance at special schools in more affluent countries, especially those with a history of segregation. The article describes how sport was used to bring together students from special schools with their non-disabled peers in neighboring mainstream schools based around the Special Olympics Unified Champion Schools^®^ program. A process evaluation was undertaken by the first author using interviews and questionnaires with 21 Special Olympics personnel and teachers from both mainstream and special schools. Based on their direct experiences, a process model was developed that could assist other sports organizations and schools to implement similar initiatives to advance the social inclusion of students with intellectual disabilities. Moreover the health outcomes from sports could be further enhanced if people with intellectual disabilities had greater access and inclusion in public health and primary care services. The process model outlined here might well be adapted to promote equity of access to healthcare which remains sadly lacking internationally.

## 1. Introduction

Access to state funded education has been achieved for nearly all the world’s children. In more affluent countries this can extend from two to 22 years of age although in poorer nations it tends to focus on those aged from five to 12 years. Schools provide children with many benefits beyond academic achievements. They contribute to their students’ social, emotional, and moral development as well as their physical health and wellbeing. They are an important partner in public health initiatives and health promotion campaigns [1].

Internationally however, various groups of children have been excluded from attending state schools, such as girls, ethnic minorities, and those with disabilities, foremost of which are those with intellectual or cognitive impairments [2]. Until recently those assessed as having low intelligence were deemed to be ‘ineducable’ and were legally excluded from schools, even in the richest countries like the United States and the United Kingdom [3]. Instead some may have attended centers organized by charities or by health services but many remained at home with family carers which continues to this day in many low and middle income countries [4]. Consequently they missed out on the benefits of school attendance.

Children with mainly physical, visual, and hearing impairments were also excluded from the schools attended by their non-disabled peers. Instead they were offered places in special schools, so that the teaching could be adapted to their particular needs [5]. Different schools specialized in educating deaf students, others for blind children and those with physical disabilities and chronic ill-health. Such special schools where located in cities so boarding arrangements were required for students whose families lived in the surrounding towns or countryside. Hence these pupils were further isolated from their families and communities. The spread internationally of special schools was helped by the colonization ethos and practices of Europeans, such as the British Empire [6] or the spread of political systems such as communism [7].

From the 1960s onwards, the inequity of the exclusion of children with disabilities from schools was highlighted in high income nations by advocacy groups, led mostly by parents. In particular they lobbied for children with intellectual and developmental disabilities to be admitted into the education systems. In response, special schools were opened for such children in many western countries and in subsequent decades in many other countries internationally. Their right to education was further ratified in the United Nations Convention of the Rights of the Child (1989: Article 23) [8] and later the Convention on the Rights of Persons with Disabilities (2006: Article 24) [9]. Both conventions have been ratified by over 190 countries.

The accepted wisdom of relying on special schools to provide equitable access to education for children with disabilities began to be questioned more widely. For example, UNCRPD stated in Article 24 (b): “Persons with disabilities can access an inclusive, quality and free primary education and secondary education on an equal basis with others in the communities in which they live”.

An especially compelling argument for inclusive schooling is the restricted access that special schools offer to all the nation’s children with disabilities. Special schools cannot provide universal coverage so it is better to invest in the ‘ordinary’ schools that exist across all countries. Nonetheless the debate continues between advocates for and against special schooling that plays out in educational policies and funding of schools [10].

One pragmatic approach is to refocus on the needs of students, both those with and without intellectual disabilities, and for this to happen initially at a local level, through building bridges between special and neighboring mainstream schools. Moreover the context initially would be around non-academic activities, such as sports. To that end, this paper describes a novel approach adopted by Special Olympics (SO); an international organization that has a presence in 172 countries to promote sports for persons with intellectual disabilities. The insights gained from their Unified Champion Schools initiative would have wider applicability beyond education, for example in community development and public health initiatives aimed at encouraging the inclusion of marginalized groups.

### 1.1. Unified Champion Schools

Although the origins of the Special Olympics in 1968 were in providing segregated sports training and competitions for athletes with intellectual disabilities, its focus now is on inclusion. The Organization’s Global Strategic Plan envisages “an inclusive world for all, driven by the power of sport, through which people with intellectual disabilities live active, healthy and fulfilling lives” [11]. One means for doing this is through the promotion of Special Olympics Unified Sports^®^ in which athletes with and without intellectual disabilities are paired in various team sports for training and competitions, with soccer and basketball being among the most popular [12]. Evaluation studies in the US and in Europe have confirmed the social and emotional benefits it provides to both sets of players [13].

Unified Sports has become embedded in another inclusion initiative of Special Olympics known as Unified Champion Schools [14]. In the USA, Unified Champion Schools focuses on ordinary schools who have pupils with intellectual disabilities enrolled in them. The aim was to further the inclusion of these students in the school community in three main ways: 1. by creating Unified Sports within the school curriculum or as out-of-school activities; 2. nurturing youth leaders among students, through membership of school councils for example; and 3. organizing whole-school activities to further social integration. Thus far, Unified Champion Schools has been rolled out in over 5000 schools across the USA with positive impacts demonstrated on the social and emotional wellbeing of the participating students and the increased support of school staff to continue and extend these initiatives [15].

A funding opportunity arose to extend Unified Champion Schools in Europe with a particular focus on countries where students with intellectual disabilities mainly attended special schools. Three national SO programs—Poland, Slovakia, and Romania—successfully bid for funds that would enable them to explore how special schools could be linked with local mainstream schools so that both sets of students and their teachers would have the experience of being included together through sports. This paper describes how the project was implemented, the lessons learnt, and the outcomes achieved. It draws on interviews and questionnaires with 21 SO personnel and teachers in order to capture their first-hand experiences of the processes that had been used in the Unified Champions Schools within each country.

### 1.2. Aims of the Evaluation

- To describe the processes used to build links between special and mainstream schools in Poland, Romania, and Slovakia.
- To identity the barriers encountered and how they could be overcome.
- To explore the reported impact on students, teachers, and schools using qualitative methodologies.

The practical guidance to emerge from these experiences would be summarized in a process model which could assist other schools in these countries and beyond, who aim to develop greater inclusion among people with disabilities and their non-disabled peers across special and mainstream schools through sports [16]. Arguably the validity of the process model would be demonstrated by the impact it had on the participants. More broadly the model could provide guidance as to how other specialist and mainstream agencies could co-operate in meeting the needs of marginalized groups in health promotion, public health matters, and community development. In due course, future studies could attest to the model’s applicability and validity beyond these countries.

### 1.3. Country Context

#### 1.3.1. Poland

Education for children in Poland is compulsory from 6 to 18 years of age and is free in State schools. In 2024, around 6.6 million children and youth were enrolled in 43,000 schools. Students with special educational needs such as intellectual disabilities may be placed in mainstream schools if they can meet the requirements for enrolment. Otherwise they will attend special schools, or units within regular schools, where they can attend until 24 years of age. Precise data is not available but there are an estimated 2000 special schools (around 4.5% of all schools) with around 70,000 pupils attending (0.1% of total). During the communist era, many children and youth with intellectual disabilities received no education or were segregated in care centers, special schools, or institutions. Today, government policies recognize their right to education and they are supportive of inclusion. More children with special needs are now welcomed into regular schools.

#### 1.3.2. Romania

Romania was part of the Eastern Bloc States in Europe. Under the Communist Party regime, people with disabilities were forced into isolation, either in institutions or in their own homes. Subsequently, elected governments have been in power and the country became a member of the European Union in 2007. However, long-ingrained attitudes were slow to change and the patterns of isolating and excluding people with disabilities and their families have persisted.

In Romania, education is compulsory and is provided almost entirely in public schools, and free of charge through to Grade 10. The state guarantees the right to education for all people with disabilities and/or special educational needs, through either special education schools or mainstream/integrated schools. The teaching curricula, methods, education materials, assessments, and staff training are created by the Ministry of National Education. In 2018, 151 separate schools were dedicated solely to children with special educational needs and attended by almost 30,000 children with intellectual disabilities who form 1.25% of the total school population.

#### 1.3.3. Slovakia

In Slovakia, education is mandatory and predominantly offered in public schools at no cost up to 19 years old. The state should ensure the right to education for individuals with disabilities and/or special educational needs, which is provided through either special education schools or pupils can be integrated into mainstream school. In addition to students in special schools, children with specific educational needs in Slovakia also have the option of individual integration into regular schools. In 2023, there were 515 dedicated schools specifically for children with special educational needs, serving nearly 34,884 children with intellectual disabilities. In the 2022/2023 school year 42,737 students were educated through integration, including home schooling.

### 1.4. Unified Champion Schools in the Three Countries

Special Olympics has been active in Poland for 40 years, in Romania for 22 years, and in Slovakia for 29 years. In each country, Special Olympics clubs had started mostly linked with special schools but relying on volunteers as sports coaches although some were teachers from the school. The grant funding for the Unified Champion Schools project came from Special Olympics International who had received a generous donation from an international donor. This enabled the three country programs to extend their involvement with mainstream schools in particular. At the end of the project and during 2025, 280 schools in Romania had been involved with the Unified Champion Schools or Unified Sports, in Poland it was 82 schools, and in Slovakia, 111 schools.

The findings section will give further details as to how the UCS project was implemented in the three countries following broadly the same strategies developed by SO in the USA but with adjustments depending on local circumstances. The projects began in 2020 in Romania just as schools closed because of COVID-19 and the first stages involved online activities in which students from both special and mainstream schools took part. Poland and Slovakia joined in 2023, which was the year when the UCS program was fully operational in the three countries, and it ended in December, 2024.

## 2. Evaluation Methodology

A qualitative methodology within a process evaluation approach [16] was used to gather first-hand information from selected informants who had direct involvement with the project from its inception and who were available to participate in the study. The main informants were Special Olympics (SO) personnel in the national program and teachers from the participating schools in the three countries: Poland, Romania and Slovakia. A previous study had obtained details of the impact on students using quantitative data and they were not included in this study [14]. SO personnel identified a sample of 17 schools where the project had been successfully implemented across the three countries. The chosen schools included the range of schools and locations in each country. They felt these schools would better illustrate the implementation of the project and how challenges encountered had been overcome.

An information sheet about the evaluation along with a consent form were prepared and made available in local languages. Interviews were conducted online by the first author using Microsoft Teams. He had no prior involvement with the project. All the informants opted to use English during the interviews. The questions asked covered the actions taken to prepare and commence the contacts between schools as well as the activities devised to promote interactions among students and teachers and to build the sustainability of the contacts. Information was sought on the barriers encountered and how they were overcome as well as the impact these experiences had on students, teachers, and the wider school community. The interview transcriptions were reviewed by the informants who made corrections and additions as required. A total of six interviews were conducted (three with SO personnel and three with teachers; one from a special school and two from mainstream schools). Together these lasted 227 min. In addition, written response came from another SO senior manager.

Based on these responses, a self-completion questionnaire consisting of eight open questions was prepared covering the topics used in the interviews. These were emailed to teachers from the schools who had been involved with the project. Teachers gave responses in local languages which were translated into English by SO personnel. In all, 14 completed the two-page questionnaire making a total of 21 respondents who had contributed their experiences. It was evident that data saturation had been achieved as no new themes were evident in the last three questionnaires returned.

Thematic content analyses were undertaken of the interviews and questionnaire responses which were taken together as the same experiences reported from two different perspectives. ChatGPT-5 (AI)—which is free to use—assisted these analyses. In part, this was to assess the role that AI could play in assisting practitioners to undertake evaluations based on qualitative information. This involved anonymizing all the interview transcripts and questionnaire responses, redacting the names of people, schools, or countries. The developers who invented ChatGPT (https://openai.com/index/introducing-gpt-5/ (accessed on 14 February 2026) describe its approach to analysis of qualitative data as being very similar to when humans make a thematic content analysis of what people say or have written down [17]. This begins by listing the various statements made by the respondents based on words, phrases, and sentences used. The lists are then interrogated to group items into common themes. Nevertheless the first two authors—who had no direct involvement with the project—checked and adjusted the grouping of items in the initial analyses prepared by AI which had proposed 12 themes. This involved combining some of the initial themes listed into process-related themes in keeping with the aims of the study. Moreover the other three authors then cross-checked the validity of the themes against their broader experiences with other schools who were not part of the study. They also approved the process model that had evolved from the analysis and the quotations used to illustrate the main themes in the process that had guided the implementation of the inclusion project. These steps helped to assure the credibility of the model, at least in the context of this sports project.

## 3. Procedures

### Participants

The managers of the projects in each country were employed by the national Special Olympics in their country. They had been in that post for an average of 15 years and came from a background in sports and education. Grant funding enabled them to second other national SO staff or employ persons on temporary contracts to liaise with schools and undertake the administrative tasks involved in bringing the students and teachers together from different schools.

Of the sample of 17 teachers who took part, seven came from special schools and 10 from mainstream schools. Overall, seven were sports/physical education teachers; seven were subject teachers and three had the role of school counselors. In all, 13 were female and four males; 13 had taught in schools for 10 or more years (some of whom had taught up to 22 years), while two had worked for between 5 to 9 years and two for between 0 to 4 years). In sum, most were experienced teachers with a range of roles within both special and mainstream schools.

## 4. The Experiences of SO Personnel and Teachers

The themes and subthemes identified by the SO personnel and those by the teachers were somewhat different in content and emphasis. Each group came from a different stand point with differing experiences, roles and responsibilities. However there was less variation across the three countries which was not surprising as they had followed the UCS program as the template. Hence, when taken together, the informants’ experience and insights enabled the creation of a process model for building inclusion across special and mainstream schools based on their shared experiences across three countries.

**Figure 1:**
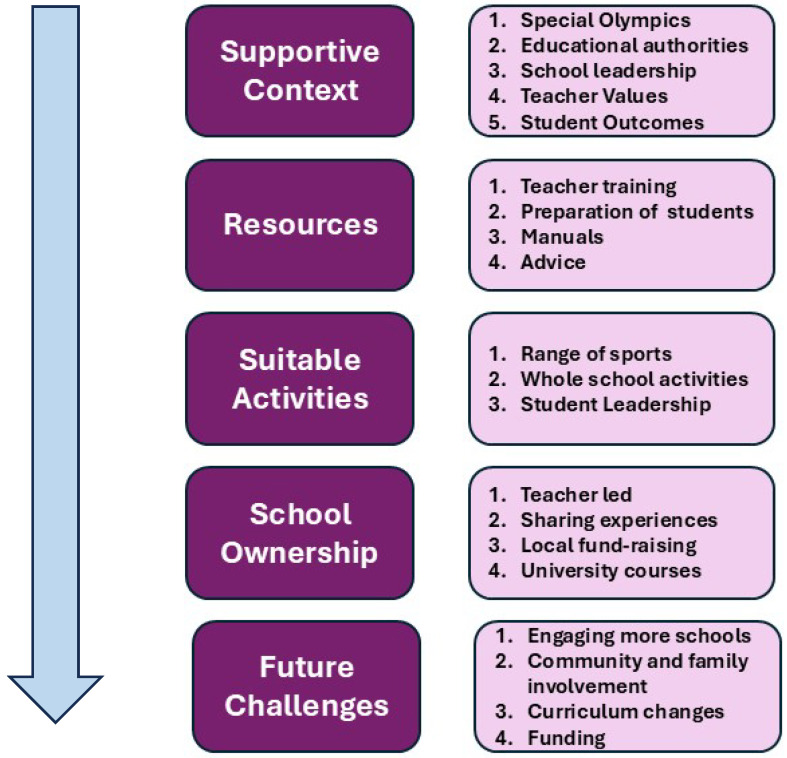
The process involved in linking special and mainstream schools through sports.

Figure 1 recasts the main themes derived from the content analysis into a model consisting of five key processes that contributed to the successful implementation of UCS in these countries along with examples of the main actions and activities that had been undertaken.

The main themes are listed broadly in the order in which they occurred as the project progressed but some needed to be actioned concurrently. Although they are shown separately, the main themes overlapped and interacted with one another over time.

For each of the themes and subthemes noted in the figure, examples are given below of the activities undertaken by the schools with support from SO personnel, using quotes from the informants as they reported examples of actions that had been taken and their outcomes. These are written in italics and a code identifying the background of the respondent is given for each. (Note: The codes are: SOI—Special Olympics personal interview; SOQ—Special Olympics written; TSI—Teacher special school interview; TSQ—Teacher special school questionnaire; TMI—Teacher mainstream school interview; TSQ—Teacher mainstream school questionnaire).

### 4.1. Theme 1: Supportive Context

Our informants recognized that a vital consideration was to assess the readiness of schools to participate in what was a novel project for the teachers and students. Indeed the ground work had taken place previously as the special schools had been involved in sports for their pupils in association with Special Olympics, although this was not the case for the mainstream schools. Also important was obtaining permission from the education authorities for schools to engage with the project and to have supportive leadership from school directors and from teachers—both class and subject teachers as well as specialist Physical Education teachers—who were disposed to encouraging curriculum innovation and community engagement. Moreover the values held by the teachers that are central to inclusion—notably equality of opportunity and respect for diversity—need to be evident in their current practice and as aspirations to motivate their activities. Recognition of the potential benefits to the students in terms of their social and emotional growth was a further requisite.

In the absence of these elements, it would seem more difficult to instigate and sustain efforts at inclusion and it may be premature to attempt to do so. Instead the focus may need to be on creating the conditions for inclusion to emerge from within the schools rather than it being imposed on them.

Here are the reflections from our informants on the five subthemes.

#### 4.1.1. Special Olympics

Special Olympics national personnel were the initial drivers of the project. They had already built relationships with special schools and through them with teachers in mainstream schools. They were well known in each country through media reports of the sports events they had organized and the success of their athletes in national and international competitions. They had prepared and distributed written and audio-visual materials to guide coaches/teachers in preparing students with intellectual disabilities to take part in sports. The availability of funding meant they could extend their sporting activities to mainstream schools. Various strategies were used by SO to recruit and prepare the schools such using their existing networks, providing information and training sessions for teachers and university students, as well as donating sports equipment and providing sports coaches. The following quotes illustrate the varying contributions made by SO personnel. In sum, the initiative came from special schools reaching out to mainstream schools and not vice versa.

*We started with our Special Olympics clubs, which very often are in the special schools …and we chose special schools from very small cities, very small villages, using their friendship, using their connections. It’s easier than in the biggest cities (with) so many other attractions. We tried to combine the schools into pairs just to be sure that two schools were quite near in the same neighbourhoods in the same society. We (held) Sports Day when children pupil students can spend time together doing sport activities, very simple, very funny, with healthy food.*

(SOI1)

*We asked NGOs (Non-Governmental Organisations) or Institutions that had collaborated before with Special Olympics and ask them if they know Schools or teachers or directors. (Also) I looked on the Internet and I chose the schools, big schools in the town and I sent details about the Information seminars we were holding to the Director by e-mail because it’s not easy to speak or to find the director. There were schools that said no because they had a lot of projects and they don’t have time to be involved in a new project. But other middle and high schools each sent two sport teachers and five tutoring teachers. Those schools who then wanted to join, we signed a partnership agreement with them.*

(SOI2)

*We started working with the universities, because we realised that we had to educate the coaches first … The teachers they were being educated during the Communist time … the teaching of or awareness of people with intellectual disability was, you know, below nothing. Those teachers who are working in schools they are still educated by this regime. We are teaching at two universities and three faculties… the students, who are going to become special educators or who are going to become coaches. And now, for example, I have six students. They are going to help us during the summer as assistants in an inclusive sports camp. They already know what coaching of athlete with intellectual disabilities is about, they will not be afraid. They lost the fear and I think this is the future of our unified (sports) because we need to spread out the new teachers, the new mentoring and teaching Ideas into them.*

(SOI3)

*The launch of the project in our school and the partner mainstream school was supported by the provision (by SO) of sports equipment necessary for the activities and rewards for students, which increased their motivation to participate and engage in the project. Additionally, the assistance of specialized coaches in various sports disciplines (gymnastics, unified bocce, unified basketball) was a real help in conducting the activities.*

(TSQ4)

#### 4.1.2. The Approval from Education Authorities

From the outset, SO programs engaged with the education authorities to obtain their permission to approach and work with schools on the UCS project. Their reputation and past experiences with schools probably contributed to a favorable response but even so, they sought a formal Memorandum of Understanding (MOU) with the education authority or other partnering bodies that outlined what each party would contribute to the UCS project. No funding was sought but the ground work was laid for the future when funding might be needed to extend and sustain the project in future years.

*The MOU was crucial for the implementation of the Project, as it allowed our organization to organize activities in schools, being endorsed by the Ministry of Education. We had formal partnerships with local NGOs and Lions Clubs … These helped us to build our UCS network and strengthen it at local level, even when the project ends.*

(SOI2)

#### 4.1.3. School Leadership

The support from the Director of schools and teachers in both special and mainstream schools was essential.

*Special Olympics wants to create a very good cooperation with the other schools. But everything depends on people. Some directors, the teachers from special schools from special institutions are not open for building the relation with regular mainstream schools.*

(SOI1)

*The passion and support from our school leaders and the enthusiastic involvement of volunteers were instrumental in getting the project underway. Their backing has been truly inspiring.*

(TSQ02)

*The project blossomed with the incredible support from passionate teachers, excited parents, and the unwavering commitment of the school administration. Their dedication has been nothing short of inspiring.*

(TMQ4)

*The project was initiated with the help of a supportive school administration and staff, collaboration with local sports clubs and organizations, and enthusiastic involvement from Special Olympics.*

(TMQ2)

#### 4.1.4. Teacher Values

The personal qualities of teachers and the school administrators were also key ingredients to building inclusion within and across schools.

*I think that the teacher needs to love what they do and I saw that our teachers do a lot of things, not only in the school program, but they do this type of activities as extra-curricular activities and that’s mean a lot. They do with their heart and they feel that it’s something important for the school to develop at this time, the social, emotional abilities to their students.*

(SOI2)

*I think that we have to love people because if we don’t love people, we cannot do this. When you love people, you want to do more for humanity, for people. So it’s something inside of everyone. I was lucky to work with these children because they teach me so much. The lesson of love.*

(TMI1)

*We participated alongside the high school teaching staff in the Project’s activities to cultivate inclusive attitudes among students, rooted in respect, understanding and acceptance of diversity, tolerance, and improving classroom relationship.*

(SOI1)

#### 4.1.5. Student Outcomes

The benefits that teachers perceived for their students—and the wider school community—from taking part in the inclusion project was another reason for schools participating in it.

*The UCS project is fantastic for our students, boosting their skills, confidence, and social abilities. It also fosters a welcoming atmosphere in our school, where each child feels appreciated and included through sport.*

(TSQ6)

*It was especially useful for students in mainstream (inclusive) schools to better understand what disability means, how to appropriately integrate a peer with special educational needs (SEN), and how to show understanding and empathy.*

(TMQ4)

*I believe it benefits pupils by building their social skills and self-esteem, promoting a sense of belonging and acceptance, and encouraging leadership and collaboration skills. For the school, it cultivates a positive and inclusive culture and enhances its reputation as a community-focused institution.*

(TSM5)

*The participating students had the opportunity to interact with people with disabilities, becoming more empathetic, more tolerant, and more understanding of the special needs of those around them, including classmates with special educational needs.*

(TMQ2)

*The students were building lasting friendships beyond sports and teachers finding new ways to engage students in the classroom. As a school we now have this UCS information in our school information bulletin.*

(TSQ1)

### 4.2. Theme 2: Resources

Translating ideas into practice means having tangible resources to give teachers the knowledge, skills, and means for creating inclusive environments for their students. This involved the provision of a range of training courses for teachers, strategies for preparing students from the two schools to meet and interact with one another, the preparation of resource manuals for use by teachers of activities they could use, and the availability to obtain advice if requested.

#### 4.2.1. Teacher Training

A variety of courses were developed by SO national organizations to prepare the teachers in both special and mainstream schools for inclusion, and also lesson plans for use with students.

*We created a seminar for the special school teachers, for them to get the knowledge about Special Olympics and about creating the Special Olympics clubs etc. And very soon we did it for mainstream school teachers to create inclusion. We raised the money from the Education Ministry and (its delivered) in their education centres. We have 16 regions and in the 16 regions we did the seminars twice a year for many, many people. They come to the Centre for a one day course minimum six hours maximum 10 h.*

(SOI1)

*We usually organize two types of seminars: one for Educators including subject teachers and school counsellors and another for Physical Education teachers. We offer diplomas/certificates for each seminar—and we sign contracts with schools—this way the schools recognize the activity of the teacher, and the teacher scores point for their annual evaluation.*

(SOI2)

#### 4.2.2. Preparation of Students

The project developed materials that teachers in mainstream schools could use to prepare their students for taking part in inclusive sports.

*The first person who go to the students is the teacher: it’s our door to the school because the teacher go to the class and tell the them about Special Olympics, about intellectual disabilities, about inclusion and how to act, how to communicate with them, people with disabilities.*

(SOI2)

*Students paid close attention during the presentations about people with disabilities and the lessons from the manual, but they showed great enthusiasm during the activities where they interacted directly with the athletes.*

(TMQ2)

#### 4.2.3. Manuals

The SO national organization prepared written materials that were given to teachers attending the seminars but also distributed to schools for other teachers to use when planning lessons and organizing sports training ad events.

*We offer to our teachers educational resources—printed manuals, ready-to-use lessons and we prepare also some PowerPoint lessons for them to implement the manual with their classes.*

(SOI2)

*The training seminars are now in the hands of the regional education centres. So every year we send them updates through emails and newsletters. When we decided to print the manuals we sent them the new books (for the seminars).*

(SOI1)

#### 4.2.4. Advice

SO personnel strived to develop a personal relationship with the teachers and maintained contact with them by phone, WhatsApp, or email to check how things were going with the schools, to answer any queries, and give advice if requested.

*We also supported them whenever they needed guidance or clarifications on how to use the resources we provided. We worked both face-to-face and online with Tutoring Teachers, PE Teachers, and Youth Leaders, offering guidance on how to prepare for different SO events they were invited to, as well as on how to use the MOU with the Ministry of Education to their school’s advantage and for their own benefit—for example, to receive extra points in their annual teacher evaluations.*

(SOQ1)

*We worked with our teachers as partners in implementation. The range of guidance requests was broad, and we tried to provide information on how to better organize Unified Sports Events and Inclusive Meetings with the Youth Leaders. We also offered teachers personalized recommendation letters if they needed them.*

(SOI2)

### 4.3. Theme 3: Suitable Activities

Careful thought was given by teachers as to the most suitable joint activities to provide for the two sets of students when they met. These included exercises and games, as well as team sports that would be mutually enjoyable. Likewise the timing and location of the meetings had to be planned, which also entailed close liaison between teachers from the two schools. The teachers also encouraged certain students to undertake leadership roles and to become advocates for their project and thereby engage the wider school communities and parents in knowing about and supporting the project.

#### 4.3.1. Range of Sports

Given the developmental variations across and within the two sets of students, it is especially challenging to find suitable inclusive activities that would match their competencies and preferences. Sports and physical pursuits have an advantage over other school subjects in this respect as they offer a wide diversity of activities in an enjoyable context.

*So we just create a sports day with the different kinds of activities. There were activities for young athletes. They were different stations for mobility, dexterity, jumping, throwing or kicking. They were team sports activities, a lot of parachute team sports activities, you know, like with the young athletes. And there is definitely football. Yes, football is strong in (our country). They have tennis, table tennis.*

(SOI3)

*The pupils found Unified Sports events like football and floorball matches, mixed skill-building sessions that foster partnership, and social gatherings, awarding, clapping and team-building exercises most helpful and enjoyable.*

(TSQ1)

*Awarding medals and trophies to children following competitions (recognizing their efforts) was the central element in motivating them to attend the organized training sessions.*

(TMQ3)

*Many of the schools organize once a month, something like Sports Day and they just join together for sports training and if they would like, they can stay for the longer time for joining the team for next weeks or months. So it depends on the children and the teachers.*

(SOI3)

*Most of the teachers, their physical educational teachers, try to organize many unified activities when they can spend time together and creating Unified Sports teams (mixed students from special and mainstream schools).*

(SOI1)

#### 4.3.2. Whole School Activities

Within each school, selected classes/year groups took part in project. However a wider aim was to make the whole school community aware of the project and in so doing, change their attitudes and perceptions of students with disabilities.

*The whole school embraced the project through vibrant assemblies, heartwarming stories shared with enthusiasm of our young athletes. (We created) Special Olympics web page too.*

(TMQ8)

*We promoted the activities within the teachers’ lounge, involving as many teaching staff as possible. We also promoted the events on the high school’s Facebook page.*

(TSQ1)

*We have some essay contests, students wrote essays about Inclusion. They make a promise that they will be inclusive. We have a Facebook page where you can see all of our inclusion activities and the promises of the students to be more inclusive and to work together.*

(TMI1)

*The project had a positive impact on school culture almost immediately, with students building lasting friendships beyond sports and teachers finding new ways to engage students in the classroom.*

(TSQ5)

#### 4.3.3. Student Leadership

The project aimed to recruit students from the schools as youth leaders so that their views and experiences would help guide the events and activities offered by the project as well as advocating for the project with the wider school community.

*The teachers are those who invite their students when we organized Youth Leadership seminars. We offer them the possibility for them to ask 2, 3 or 4—it depends on the county—to participate in our local Youth leadership seminar. The seminar was the opportunity for us to meet the students and to prepare them to be leaders. We told them about what it means to be a leader for inclusion.*

(SOI2)

*We have assistant coaches. This is our Youth Leadership. The young students from the private schools. (They come) after school, it’s during our training lessons. They are doing assistant coaches in track and field like athletics preparation; swimming classes with young athletes and also artistic gymnastics.*

(SOI3)

*We organize 5 or 6 Regional Youth Leader seminars, workshops. We try to find pairs when athlete and one partner together from the (neighbouring) schools, come to the workshop where they learn how to be the messenger, how to get the knowledge about Special Olympics and how to be the leader in volunteering and creating the Youth Leaders group in their school.*

(SOI1)

### 4.4. Theme 4: School Ownership

A major outcome from the project was that schools would take ownership of it and continue to build inclusion between the two schools. SO personnel would still be available to support their efforts while also working nationally to ensure that teachers were better trained to make mainstream schools more inclusive of pupils with additional needs.

#### 4.4.1. Teacher Led

From the beginning, teachers were empowered to take on leadership of the project within their schools albeit with support from SO personnel, but the need for this was reduced as the project unfolded. The aim was to reduce a dependency on ‘outsiders’ while ensuring that teachers and schools would sustain the project from their own resources.

*Schools have to get involved because the activities differ from traditional ones conducted in the classroom. They involve resources, attitudes, and values that are essential to promote among students as life skills.*

(TSQ1)

*Because supporting students with SEN (Special Educational Needs)—and especially understanding them—has become a priority in education, participation in the Unified Champion Schools projects offers real support in this regard, bringing students with and without disabilities closer together. Through the support teacher in the inclusive school; through collaboration and by highlighting the purpose of the project.*

(TMQ4)

*We empower teachers to organize their own activities in the school or in the community. And I think that they are more prepared to organize this type of activities because after the seminar they know more about how to be a leader and how to Implement an activity.*

(SOI2)

*There are some counties and some towns where teachers started to organize their own local competition … with a Unified Sports team from two schools, a special school and a mainstream schools.*

(SOI2)

#### 4.4.2. Sharing Experiences

From the outset, teachers were encouraged to share their experiences with colleagues in and across the participating schools. Information technology greatly assisted this.

*I saw the community of teachers grow and we had also a teacher council and teachers from different counties started to make a WhatsApp group and they put (information about) what they did in their school for the basketball week. And also it was interesting that they plan flash mode dance in the same day or in different counties. They speak together and they plan to do common activities.*

(SOI2)

*We have now a lot of online tools that help us to spread the news and to involve them. Conferences, seminars in which you can share good practices from our country and other countries that will get involved in this amazing Project. I am so stunned when I think that they start and they grow so fast and in such a beautiful way. So I think that these are our first tools. I think that in every country, Special Olympics have some teachers that are involved in this. And they can be may be the promoters in every country with your help, of course in in conference and seminars and sessions.*

(TMI1)

#### 4.4.3. Local Fund-Raising

There are costs involved in linking the schools, primarily travelling to events (although in cities, public transport could be used), the purchase of equipment, refreshments, and medals and certificates for students. Schools were given encouragement and support to raise donations locally from charities including in-kind donations from businesses.

*Last year we start some fundraising to help Special Olympics sport teams to go to the international competition and to teach them how to not only to get help and to help the others.*

(TM1)

*We partnered with their school projects and guided them through small fundraising events in their schools. We provided visual and branding feedback when teachers needed to organize events in their schools and wanted to personalize small giveaways for their students.*

(SOI2)

#### 4.4.4. University Courses

A longer term initiative to support school ownership was to ensure that teachers were better prepared during their university training for the inclusion of students with disabilities. One national SO program described their approach.

*We are teaching at two universities and three faculties, teaching two hours a week. We are teaching about special Olympics and about inclusive sports. And we are teaching the university students who are going to become special educators or who are going to become coaches. So, this is the idea: to start with educating the future, the young generation, because they are going to teach the children.*

(SOI3)

### 4.5. Theme 5: Future Challenges

Informants were asked to identify the future challenges likely to arise locally for them as well as promoting inclusion across their respective countries. In part, this is forewarning that will assist in the planning for addressing these challenges. Their responses are noted below and echo those reported in the literature on social inclusion.

#### 4.5.1. Engaging More Schools

*I would advise other schools to start small with pilot program and gather feedback, engage with local community partners for resources and support, and foster an inclusive mindset among staff and students. We can guide them how to start.*

(TSQ5)

*Promoting our project on our Social Media and also through local press, more schools hear about our achievement and call our office to get involved. Also this year we have added our events in the formal calendar of the Ministry of Education, as that it is a real incentive for our new schools. Growth can be attained and maintained, only gradually and locally. This is our belief.*

(SOQ1)

#### 4.5.2. Community and Family Involvement

*We organize many family seminars just to be sure that they understand the Special Olympics idea that they understand the athletes participation.*

(SOI1)

*Involving family members was a challenge in our country, mainly because our partnership was with the schools; we did not have access to parents. Maybe in the future we could add a parent component in our educational projects, as it is important to reach directly to family members of students in the mainstream schools, from our point of view. The students from the special schools are mainly from disadvantaged families, or live in state institutions without parents. This is why they have difficulties to meet with teachers, not to mention to come to SO events. Working with families in school environments it is difficult but not impossible if we add a component like this in a future project application.*

(SOI3)

#### 4.5.3. Curriculum Changes

*I think that we have to do changes maybe in the curricula of schools to have maybe a new subject named “Education for inclusion and diversity” because then we will have enough time, even if we have one hour per week in which we teach this education.*

(TMI1)

*We think that attitude change in schools comes from curricula changes. Our three manual resources help but in order to reach systemic change we would need to take the approval of the Ministry of Education and to transform the curricula in a national subject to be taught nationally. This initiative would not be possible without financial support from the State—Ministry of Education. We think this is a medium to long term plan as the current uncertain situation with our Government and the financial restrictions and cuts in human resources, would affect this plan starting in the next two years.*

(SOQ1)

#### 4.5.4. Funding

*The schools want SO to travel to their area and make events there. For this we need to have money to go there, also some budget too if they want to have medals or equipment. It’s important to have the budget for the project to continue to offer them this type of small things for the activities in schools.*

(SOI2)

*The money for the Inclusion project has been cut. Special Olympics has its own budget for national activities and it’s up to us how we divide the budget. We will continue to provide schools with monies.*

(SOI4)

*I think funding has to be found by our SO organization either in the private sector, or through different financing program from SO internationally. The partnership with the Ministry of Education will continue to remain as it is, without involving the Ministry from a financial point of view. Their endorsing our activities through the MOU, gives us access to sports venues and most importantly to schools. We need to continue to be flexible and to invent a lot in order to attract financing of our organization.*

(SOQ1)

### 4.6. Reflections on Improvements

In the interviews and questionnaires, teachers were also asked to reflect on possible improvements to the Project but they were less forthcoming with ideas. Those who did, focused on extending the support offered to schools and engaging more schools in it. Mention was also made of having greater engagement with parents, and encouraging students to meet away from schools.

*Additional support like more funding for specialized equipment, further training for teachers on inclusive coaching methods, and regular workshops on inclusion would have improved the project.*

(SOI3)

*Our only regret is that we would like to see more activities, involving as many students and teachers as possible, to help them understand disability as an opportunity to become better people, not as a burden on the school.*

(TQ4)

*Many parents even don’t know that their children participate in so many workshops and sports. It is very challenging. We really have to make a big education and awareness campaigns through the societies for families.*

(SOI1)

*Although the students are more appropriate, they don’t ignore each other (now) they play together and do sport with each other. But I think that’s only in school, because also for typical students, they don’t have time to spend together. They go home and not all the students with disabilities have phones and the possibility to meet because they are not in the same street.*

(TMI2)

### 4.7. Activities That the Students Found Most Helpful and Enjoyable

Our informants were also asked to give their perceptions of the activities that the students in both sets of schools had found most helpful and enjoyable. Among the more common responses were the sports played, the experience of being on a team, the social gatherings, and the award of medals at the competitions.

*The swimming sessions and football matches have been pure joy for the pupils, offering them a chance to shine, learn, and grow in ways we never imagined.*

(TMQ4)

*They enjoyed the sports days with different sports, the joint workouts in training sessions and volunteering at competitions.*

(TSQ2)

*Awarding medals and trophies to children following competitions (recognizing their efforts) was the central element in motivating them to attend the organized training sessions.*

(TSQ2)

*The pupils found Unified Sports events like football and floorball matches, mixed skill-building sessions that foster partnership, and social gatherings, awarding and clapping and team-building exercises most helpful and enjoyable.*

(TMQ5)

They spoke too about the students’ reaction to the activities they had organized to engage the whole school community.

*We shared our journey and the project’s success through school meetings, newsletters, and by celebrating milestones with the entire school.*

(TSQ2)

*The Unified Games and Thematic Days draw the attention of the entire school to the importance of diversity and contribute to a positive school culture.*

(TMQ4)

*We promoted the activities within the teachers’ lounge, involving as many teaching staff as possible. We also promoted the events on the high school’s Facebook page.*

(TMI2)

*As a result of the Unified Champion School activities: students and teachers in mainstream schools became more empathetic and more engaged in the education and support of students with SEN.*

(TMQ2)

### 4.8. Advice from Teachers to Other Schools

Finally, the teachers were asked to give advice to other schools who were considering linkages between special and mainstream schools. Here are some of their responses.

*I would advise other schools to start small with pilot program and gather feedback, engage with local community partners for resources and support, and foster an inclusive mindset among staff and students. Special Olympics can guide them how to start.*

(TSQ5)

*I would like to express special appreciation for the way the project coordinators were involved. We consistently received information, and they organized all activities with great attention to detail.*

(TMQ2)

*I would encourage them to start small, involve the community, and celebrate every success along the way. It’s a rewarding journey that brings joy and growth to everyone involved thanks also through Special Olympics channels.*

(TSQ5)

*Schools can get involved because the activities differ from traditional ones conducted in the classroom. They involve resources, attitudes, and values that are essential to promote among students as life skills.*

(TSQ1)

*Participation and involvement in the project can bring benefits primarily to children with disabilities, students in mainstream education, parents, and participating teachers.*

(TMQ7)

## 5. Discussion

This small scale study had a number of strengths; notably it combined the perspective of people delivering the project and the principal recipients of it. The project took place in three different countries, which helps to confirm its applicability to other nations with a strong tradition of special schooling. It also confirms the role that non-academic subjects such as sports can play in nurturing the social and emotional development of students and creating inclusive environments. By focusing on the processes and strategies that were used, insights can be offered as to how children and people with disabilities could become more included in other community initiatives such as public health.

That said, the evaluation had its limitations. The sample was drawn from selected schools who were already well disposed to the project’s ambitions. Different and arguably more challenging issues may arise with schools who would be reluctant partners with other schools. That said, our informants have described some of the features that were needed in preparation for implementing similar projects although their further development needs to be researched. Insights from implementation science provide useful guidance in identifying priority topics for further studies [18].

We had limited insights into how other stakeholders perceived the project, particularly the students who had participated in it. Gaining access to the students from education authorities and obtaining parental or guardians’ permissions would have been too daunting given our limited resources for the evaluation, Fortuitously though, over 200 students from both sets of schools in Romania had participated in a prior study that used questionnaires and rating scales to assess the impact on them [14]. Nearly all students with intellectual disabilities enjoyed the UCS activities, felt respected and accepted, and wished to be part of these activities in the future. They reported that the school climate had become more inclusive; their peers were kinder to one another (bullying was diminished) and the feeling of belonging in school was enhanced. Likewise, students in mainstream schools reported that empathy and consideration towards their fellows with intellectual disabilities had increased and that their interactions with these students had changed for the better. Higher levels of social–emotional skills were found after the project. These echo the outcomes reported from the USA [15].

This study adds to the growing literature that documents the rationale for how sports can make a contribution to creating a more inclusive community for people with intellectual disability. The justification is rooted in the Convention on the Rights of Persons with Disabilities (United Nations: Article 30.5a) [9] which states: “to encourage and promote the participation, to the fullest extent possible, of persons with disabilities in mainstream sporting activities at all levels”. Moreover, a number of theoretical frameworks have been proposed to underpin interventions of which the creation of social capital through expanding social networks and community participation is a dominant theme [19,20]. These too can guide different public health initiatives, although to date this has been inadequately investigated [21].

Up to the present, less attention also has been paid to how these rights and theories can be put into practice to benefit people with intellectual disabilities. Nonetheless past studies have examined how Unified Sports has developed in the context of Special Olympics [12] and also how athletes with intellectual disabilities can become more included in community sports clubs [22]. More broadly there is a growing literature as to how other marginalized groups can be included in sporting activities [23]. However in many of these reports the focus has tended to be on the outcomes and impact on participants. For example, a recent study identified three main outcomes: social inclusion, health and well-being, and personal development and growth [24]. The physical health benefits included improved fitness and reduced chronic diseases, with mental health improvements such as stress relief and anxiety reduction. Personal development included leadership skills, teamwork, and providing educational and career opportunities.

The added value of the present study is the identification of strategies to advance the process of inclusion by teachers and sports personnel. These are described in terms of five main themes and subthemes within each. Moreover the quotations presented give specific examples that they could consider when planning similar schemes. Further information and useful resources are available on the Special Olympics website [25].

The history of certain nations and their past political systems can leave a long lasting legacy of social exclusion for marginalized groups as was the situation in the participating countries in this study [26]. The applicability of the strategies used by Special Olympics to the use of sports with other marginalized groups is worthy of further investigation across countries internationally, when different cultural and systems issues may arise [23]. Moreover future studies could usefully identify the applicability of the strategies identified in terms of sporting initiatives to other aspects of people’s lives such as employment, housing, and access to healthcare [27]. Indeed the health outcomes from sports could be further enhanced if people with intellectual disabilities had greater access and inclusion in public health and primary care services. The process model outlined here might well be adapted to promote equity of access to healthcare which WHO reports is sadly lacking internationally [28].

## 6. Conclusions

The social inclusion of people with intellectual disabilities remains a challenge in many nations. Often, the specialized and segregated services provided to them in childhood can inadvertently contribute to their exclusion. This study demonstrates the social gains that can be obtained by linking special and mainstream schools through sports. But this required careful planning and dedicated implementation from both sets of schools. The process evaluation undertaken in three countries in Eastern Europe identified five main themes that guided the implementation of a Unified Champion Schools project developed by Special Olympics. These have been brought together into a process model that could guide the extension of this project to other schools in and beyond Eastern Europe and more generally its use to promote the social inclusion of other marginalized groups.

## Data Availability

The anonymized data reported in this paper is available on reasonable requests to the corresponding author.
